# Single Ca^2+^-activated Cl^−^ channel currents recorded from toad olfactory cilia

**DOI:** 10.1186/s12868-016-0252-0

**Published:** 2016-04-25

**Authors:** Ricardo Delgado, Casilda V. Mura, Juan Bacigalupo

**Affiliations:** Department of Biology, Faculty of Sciences, University of Chile, 7800024 Ñuñoa, Santiago Chile

**Keywords:** Odor transduction, Olfactory cilia, Ion channel, Calcium, Olfactory sensory neuron, ClCa4l, Bestrophin-2, Anoctamin-2

## Abstract

**Background:**

Odor transduction, occurring in the chemosensory cilia of vertebrate olfactory sensory neurons, is triggered by guanosine triphosphate-coupled odor receptors and mediated by a cyclic adenosine monophosphate (cAMP) signaling cascade, where cAMP opens cationic non-selective cyclic nucleotide-gated (CNG) channels. Calcium enters through CNG gates Ca^2+^-activated Cl^−^ channels, allowing a Cl^−^ inward current that enhances the depolarization initiated by the CNG-dependent inward current. The anoctamin channel 2, ANO2, is considered the main Ca^2+^-activated Cl^−^ channel of olfactory transduction. Although Ca^2+^-activated Cl^−^ channel-dependent currents in olfactory sensory neurons were reported to be suppressed in ANO2-knockout mice, field potentials from their olfactory epithelium were only modestly diminished and their smell-dependent behavior was unaffected, suggesting the participation of additional Ca^2+^-activated Cl^−^ channel types. The Bestrophin channel 2, Best2, was also detected in mouse olfactory cilia and ClCa4l, belonging to the ClCa family of Ca^2+^-activated Cl^−^ channels, were found in rat cilia. Best2 knock-out mice present no electrophysiological or behavioral impairment, while the ClCa channels have not been functionally studied; therefore, the overall participation of all these channels in olfactory transduction remains unresolved.

**Results:**

We explored the presence of detectable Ca^2+^-activated Cl^−^ channels in toad olfactory cilia by recording from inside-out membrane patches excised from individual cilia and detected unitary Cl^−^ current events with a pronounced Ca^2+^ dependence, corresponding to 12 and 24 pS conductances, over tenfold higher than the aforementioned channels, and a approx. fivefold higher Ca^2+^ affinity (K_0.5_ = 0.38 µM). Remarkably, we observed immunoreactivity to anti-ClCa and anti-ANO2 antibodies in the olfactory cilia, suggesting a possible cooperative function of both channel type in chemotransduction.

**Conclusions:**

These results are consistent with a novel olfactory cilia channel, which might play a role in odor transduction.

## Background

 Odor transduction is confined to the chemosensory cilia of olfactory sensory neurons (OSNs), where it gives origin to a depolarizing receptor potential. Odorants bind to G-protein coupled receptors, which activate adenylyl cyclase type III by coupling to a heterotrimeric G-protein (Golf), producing cyclic-AMP. This nucleotide directly opens non-selective cationic cyclic nucleotide gated (CNG) channels [1, 2]. Ca^2+^ ions entering the cilia through these channels in turn gate Ca^2+^-activated Cl^−^ channels (CaCCs) [3], generating a Cl^−^ efflux that amplifies the depolarization initiated by the CNG-dependent current [4, 5] (but see [6]). The CNG channel has been thoroughly characterized [7], but the CaCC channel has been difficult to assess, and has been focus of attention since it was discovered [3] and its physiological importance unraveled [4, 5]. Its electrophysiological characterization has been hampered by the extremely small conductances of the reported channels and the miniscule diameter of the olfactory cilia, bordering the limit of resolution of light microscopy [8]. For this reason, only macroscopic current measurements had been performed on OSNs by means of whole cell recording [6], excised entire individual cilium [3] and patch clamping the dendritic knob [9], an apical round-shape structure of the dendrite from where the olfactory cilia emanate. As the knob has a nearly tenfold larger diameter than a cilium (~2 vs. 0.2 µm), it is more amenable for patch clamping; nevertheless, the measurements obtained from the dendritic knob assume that the same channels of the cilia are also present in the knob. No single Ca^2+^-activated Cl^−^ currents have been directly recorded from the ciliary membrane, limiting the biophysical characterization of the olfactory Cl^−^ conductance.

Anoctamin-2 (ANO2), an ion channel belonging to the anoctamin family of CaCCs, has been proposed as the main contributor to the odor-dependent Ca^2+^-activated Cl^−^ conductance, as the Cl^−^ current is largely absent in the ANO2 knock-out mice. However, field potential recordings from the olfactory epithelium of these mutant mice are only partly reduced and these animals do not exhibit impairment in smell behavioral tests [10], suggesting that more than one CaCC species might be involved in odor transduction. A second CaCC channel type was found in the olfactory cilia, Best2 from the Bestrophin family [11], later considered not to be implicated in odor transduction because its genetic ablation had no influence in smelling behavior [12]. Both channels possess estimated conductances below 1 pS, too small for resolving unitary currents. Two additional CaCCs, ClCa4l and ClCa2 from the ClCa family, were recently reported in rat olfactory cilia by PCR, western blotting and immunochemical evidence, being ClCa4l much more abundant than ClCa2 [13]; none of these two channels have been characterized electrophysiologically. Direct patch-clamp recordings from the chemosensory cilia of Ca^2+^-activated Cl^−^ currents at the single-channel level would help to clarify this scenario, but they have not been performed for any of the aforementioned channels.

Here we explored the ciliary membrane for detectable single-channel currents by recording directly from inside-out membrane patches excised from toad olfactory cilia, taking advantage of their somewhat larger dimensions than those of mammals, and found a Ca^2+^-activated Cl^−^ channel not previously described. A complementary immunocytochemistry study with anti-ClCa and anti-ANO2 antibodies revealed co-immunoreactivity in the toad cilia, raising the possibility that the novel channel may belongs to the ClCa channel family.

## Results

### Ca^2+^ dependence of a ciliary Cl^−^ channel

**Fig. 1:**
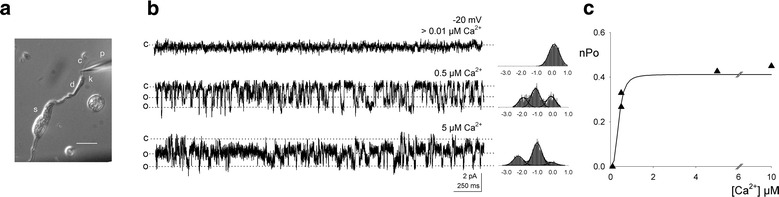
Ca^2+^ dependence of a Ca^2+^-activated Cl^−^ channel from toad olfactory cilia. **a** Isolated olfactory receptor neuron under DIC optics; s, soma; d, dendrite; k, dendritic knob; c, cilia; p, patch-clamp pipette. **b** Cl^−^ currents from an inside-out excised ciliary patch at different Ca^2+^ concentrations (V_m_ = −30 mV, V_bath_ − V_pipette_); whole-point amplitude histograms are shown by each trace. **c** Plot of nPo vs. [Ca^2+^] (nPo: number of channels times the open probability; see “Methods” section). The data point are fit with a Hill function; K_0.5_ = 0.38 µM, n = 2.7 (N = 4)

A typical dissociated toad olfactory neuron is shown in Fig. 1a in which the chemosensory cilia are clearly visible. Inside-out membrane patches excised from a cilium with very high resistance pipettes exhibited discrete Cl^−^ current events with a sharp Ca^2+^ dependence. Channel current recordings at different Ca^2+^ concentrations in the cytoplasmic side of a patch are presented in Fig. 1b. The solutions with the different Ca^2+^ concentrations tested were exchanged by perfusion of the bath. No channel activity was observed at 0.01 µM Ca^2+^, whereas a pronounced activation occurred at 0.5 µM Ca^2+^ and even higher at 5 µM. Figure 1c depicts the Ca^2+^ dependence of the channel. As the number of channels in the patch could not be determined, the ordinate was expressed as the number of channels times the open probability, nPo (“Methods” section). The data points from the experiment in which more points were collected were fit with a Hill function that determined a K_0.5_ = 0.38 µM and a Hill coefficient of 2.7 (N = 4). It is important to point out that because these experiments were extremely hard to do, not only due to the difficulty of obtaining ciliary patches but also because most membrane patches did not last long enough to allow collecting sufficient data to build reasonably complete curves.

### Current–voltage relation of the ciliary Ca^2+^-activated Cl^−^ channel

**Fig. 2:**
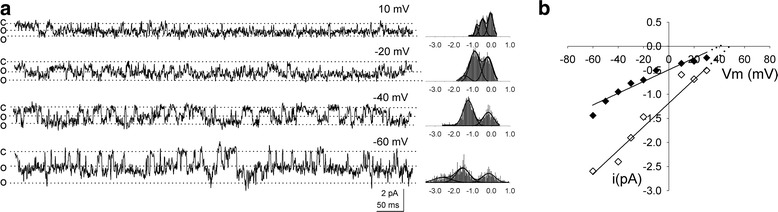
Current–voltage relation of the ciliary Ca^2+^-activated Cl^−^ channel. **a** Current traces recorded at various voltages. **b** Current–voltage *curves* from the two peak current levels in the histograms in **a** (*filled and empty diamonds*); slope conductances 12 and 24 pS. Ca^2+^ concentration: 0.5 µM. (N = 3)

Figure 2a displays single-channel current recordings at different pipette potentials, under an approximately tenfold Cl^−^ gradient (10 mM pipette/110 mM bath) and 0.5 µM Ca^2+^ in the internal side (bath). Two current–voltage curves built with data from this patch are presented (Fig. 2b; N = 3), determining slope conductances of 12 and 24 pS (filled and empty diamonds, respectively).

Immunoreactivity to a Ca^2+^-activated Cl^−^ channel of the ClCa family and to ANO2 in the olfactory cilia.

**Fig. 3:**
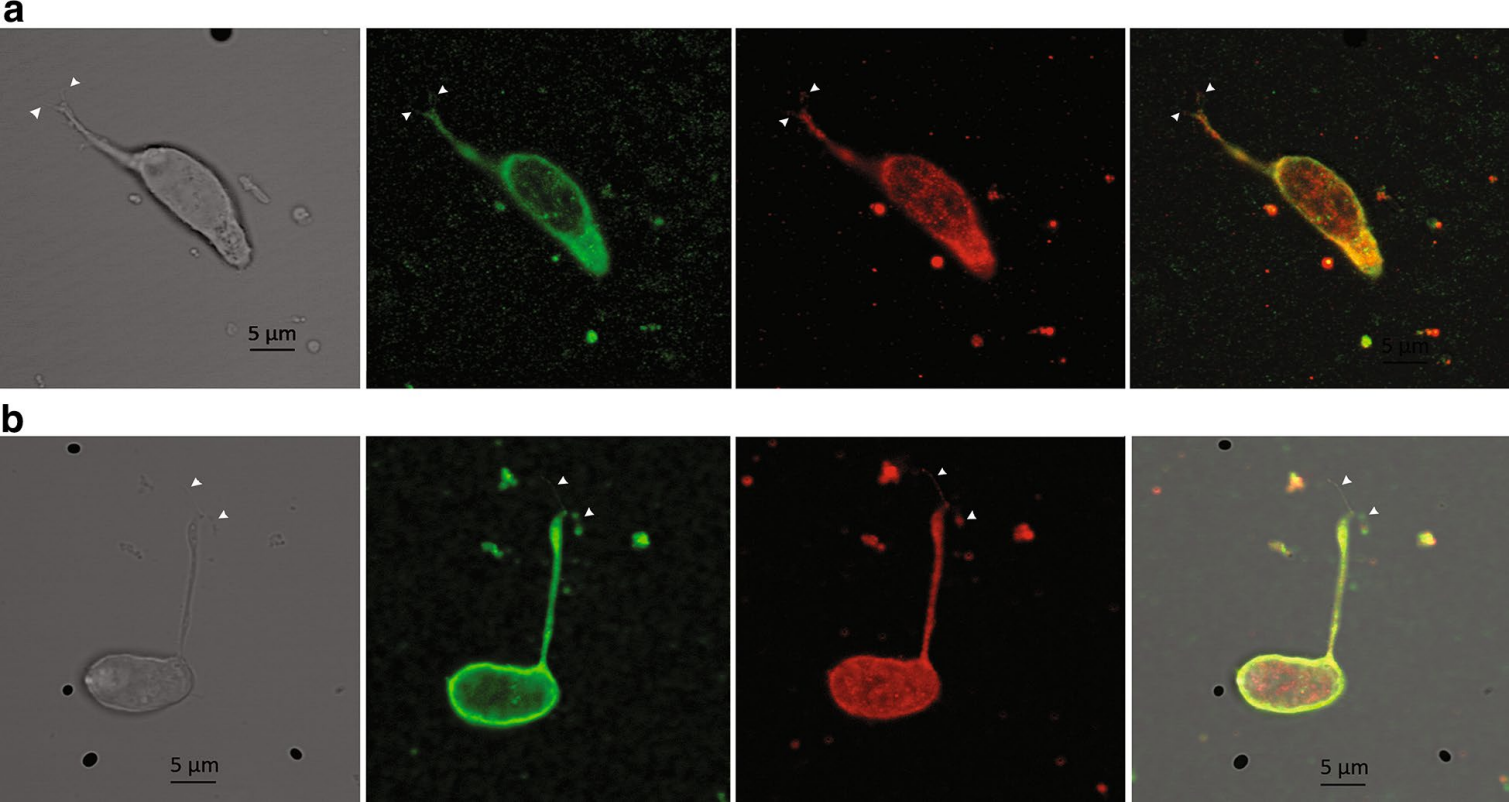
Co-expression of ClCa and ANO2 channels in isolated toad olfactory cilia. **a** Transmission image of the cell (*1st panel* on the *left*). Fluorescence images with anti-ClCa (*2nd panel*), anti-ANO2 (*3rd panel*) antibodies and merge of the two images (*4th panel*). **b** Same as in **a**, for a different cell. *Arrow heads* are pointing the cilia (N = 9)

As the only prominent CaCC besides ANO2, ANO6 and Best2 reported in olfactory cilia is ClCa4l [13], we performed immunocytochemistry on isolated OSNs with an antibody against this channel. In addition, we used an anti-ANO2 antibody to compare the expression of both channels in the cilia. Remarkably, we observed immunoreactivity to ClCa and ANO2 in all cells tested (Fig. 3a,b; N = 9).

## Discussion

In this work we are presenting the first electrophysiological recordings of a Ca^2+^-activated Cl^−^ channel directly obtained from vertebrate olfactory cilia. Based on the conserved morphological and functional characteristics of the olfactory sensory neurons between amphibians and mammals studied so far [14], and in particular the similarity of their transduction cascades, it is plausible that orthologs of the toad CaCC cilia channels are shared with mammals. Often proteins from evolutionarily distant species cross-react to antibodies developed to one of them; amphibian and mammalian CNG [14] plasma membrane Ca^2+^ ATPase [15, 16] and Na^+^/Ca^2+^ exchanger [16] of olfactory cilia illustrate this fact. [15].

The channel exhibits a steep sigmoidal dose–response relation, revealing a pronounced Ca^2+^ dependence in a narrow range of Ca^2+^ concentrations (below 0.5 µM) within physiological levels. Notably, although the curve presents a similar shape than those of ANO2 and Best2, it is shifted to lower Ca^2+^ concentrations; its Ca^2+^ affinity is considerably higher than these two other channels, as indicated by its K_0.5_ of ~0.38 µM, compared to 1.8 µM for ANO2 and 4.7 µM for Best2 [10, 11]. Previous estimates from macroscopic current measurements in excised intact frog cilia are within the same range of the two mammalian channels (γ = 0.8 pS, K_0.5_ = 4.8 µM) [17].

The evidence is consistent with a Cl^−^ channel. This channel also conducts acetate, the anion used to replace Cl^−^ in the pipette, as revealed by the ~40 mV reversal potential in the current–voltage relations. In agreement to the Goldman, Hodgkin and Katz equation, P_Ac_/P_Cl_ = 0.47, close to previously reported values for CaCCs [10].

The channel reported hereby possesses distinct properties compared with the two olfactory CaCCs previously reported in mice, namely ANO2 [10] and Best2 [11], suggesting that they correspond to a novel type of CaCC channel protein. Because of its relatively large unit conductance (γ > 10 pS), its single-channel currents could be clearly resolved; in contrast, those from the other two channels are below the resolution of patch clamp amplifiers, as their unitary conductances are in the sub-pS range, according to noise analysis estimates (0.8 and 0.26 pS, respectively [10, 11]).

The immunochemical data showing co-expression of ClCa4l and ANO2 in the cilia suggests that the toad CaCC may correspond to an ortholog of ClCa4l, but in the absence of molecular biology evidence this cannot be established. An electrophysiological correlation would have helped to support the presence of the channel reported hereby and ANO2 in the olfactory cilia such as observing the current events of the two different channels in the patches; however, this is not plausible mainly because of the size of the ANO2 unitary currents.

Why has this channel not been detected in mammals, in spite of its big conductance? Besides the trivial possibility of not being expressed in them, it is plausible that even though its single-channel currents are sufficiently large to be resolved, their overall number in the cilia could be much smaller than that of ANO2; in such a case, the ANO2 currents would dominate, masking the currents of the other channel in the macroscopic measurements.

Recordings from inside-out ciliary patches revealed transitions with complex kinetics. The data do not allow discriminating whether the recordings emerged from an individual channel with more than one conductance state or from more than one channel in the patches. Further investigation is necessary to elucidate this issue.

Our immunocytochemical evidence obtained with an anti-ClCa antibody is consistent with the presence in the toad olfactory cilia of an orthologous of the rat ClCa4l [13], which may have some role in chemotransduction. It is debated whether or not the CaCl family is comprised of functional ion channels or of accessory proteins that regulate the expression of other CaCCs [18, 19]. Further investigation is required to identify the molecular nature of the toad CaCC reported hereby and establish its functional role.

A channel with the relatively high Ca^2+^ affinity, strong Ca^2+^ dependence and comparatively large conductance such as the toad CaCC could play a relevant role in odorant responses. It is reasonable to imagine that as the CNG conductance develops, the membrane potential begins to depolarize and the Ca^2+^ level to gradually rise, opening the CaCCs. The first CaCC to open would be that with the highest Ca^2+^ affinity; the higher its conductance, the bigger would be its impact on the receptor potential. This would be the case for ClCa, which fulfills both characteristics (K_0.5_ = 0.38 µM, γ > 10 pS). The role of this channel could be to boost the receptor potential in its early phase, until Ca^2+^ levels reach the threshold for the activation of the lower Ca^2+^ affinity more abundant CaCCs, which would then gradually govern the receptor potential by allowing a relatively much larger Cl^−^ current component.

## Conclusions

The reported channel suggests that the electrophysiological tools possessed by the olfactory cilia to carry out chemotransduction may be more complex than previously considered.

## Methods

The aim of this study was to report a novel Ca^2+^-activated Cl^−^ channel discovered in olfactory cilia and explore its possible significance and identity, using electrophysiology and immunocytochemistry.

### Animals

Experiments were conducted on isolated olfactory sensory neurons (OSNs) from toad (*Caudiverbera caudiverbera*) olfactory epithelium. Olfactory epithelia were extracted from the nasal cavity and placed in a solution made of (mM): 115 NaCl, 2.5 KCl, 1 CaCl_2_, 1.5 MgCl_2_, 3 glucose and 10 HEPES; pH 7.6.

Toads were purchased from a supplier authorized by the Chilean Service of Agriculture and Livestock (Servicio Agrícola y Ganadero). Animals were anesthetized, sacrificed by sectioning the spinal cord and pithed, following the protocol used by the Ethics Committee Institutional Animal Care Committee of the University of Chile, which corresponds to the official protocol of the Fondo Nacional de Ciencia y Tecnología de Chile (Chilean National Fund for Science and Technology, CONICYT).

### Preparation

Experiments were carried out on toad rather than mammalian olfactory cilia because their bigger size makes them more amenable for patch clamping. Olfactory sensory neurons were mechanically dissociated from small pieces (~1 mm^2^) of olfactory tissue directly into the recording chamber and viewed with an OLYMPUS IX70 inverted microscope with DIC optics (60X objective, Olympus, Japan).

### Solutions

The bath solution contained (mM): 110 NMDG (N-Methyl-D-glucamine), 110 HCl, 1 EGTA, 0.7 CaCl_2_ and 10 HEPES; pH 7.0, 0.5 µM free Ca^2+^. The patch pipettes were filled with (mM): 115 NMDG, 10 HCl, 110 acetic acid, 1 EGTA, 0.7 CaCl_2_ and 10 HEPES; pH 7.0. Free [Ca^2+^] was determined with Winmaxc 2 software (http://web.stanford.edu/~cpatton/webmaxc2.htm). HCl was used to adjust the pH to 7.0.

Exchange of bath solutions was accomplished by perfusing the chamber with six volumes of the desired solution.

All chemicals were from Sigma-Aldrich, except of those specifically indicated.

### Electrophysiology

An electrophysiological characterization of the channel was approached by single-current patch-clamp recordings from the olfactory cilia. Currents were recorded with an Axopatch 200-B amplifier, digitized (Digidata 1440) to store in a PC using pClamp 10 software, also used for data analysis (all three from Molecular Devices, USA). Recording pipettes were made of borosilicate glass (Hilgenberg-GmbH, Germany) and pulled in a horizontal puller (P-1000, Sutter Instruments, USA) to tip resistances of >50 MOhm. The number of channels in the excised patches were not known, therefore what we measured was the probability times the number of channels, nP_o_ (nP_o_ = T_o_/T_o_ + T_c_). The amplitude values of the currents were measured by all-point histograms, shown by each current trace in Figs. 1 and 2.

### Immunocytochemistry

Immunofluorescence was done according to Mura et al. [20]. Briefly, mechanically dissociated OSNs were attached to coverslips pretreated with 10 µg/ml poly-l-lysine (Sigma, USA). After 3 h they were fixed for 30 min in 4 % paraformaldehyde in PBS at 4 °C and permeabilized with 0.2 % Triton in PBS at room temperature. Unspecific sites were blocked by incubation in phosphate buffer saline, PBS, containing 0.2 % gelatin-PBS. The cells were then incubated overnight with the primary antibody rabbit anti-human ANO2 (1:100; Sigma, catalog number HPA036276USA) and mouse anti-rat ClCa (1:100; custom made) [13] diluted in gelatine-PBS at 4 °C in a humidified chamber. After this incubation the cells were washed 6 times with gelatin-PBS for 30 min. Incubation with the secondary antibodies anti-rabbit AlexaFluor-546 and anti-mouse Alexa-488 (1:1000; Molecular Probes, Eugene, Oregon, USA) was then performed for 2 h at room temperature in the dark. The cells were washed five times with PBS and once with distilled water for mounting. The coverslips were mounted on glass slides with Fluoromount (Southern-Biotech, USA). The cells were viewed in an inverted laser confocal microscope (Carl Zeiss LSM 510 Meta, Germany, 63x objective).

## Abbreviations

ANO2: anoctamin 2 channel of the anoctamine family
Best2: Ca^2+^-activated Cl^−^ channel of the bestrophin family
CaCC: Ca^2+^-activated Cl^−^ channel
ClCa: Ca^2+^-activated Cl^−^ channel of the ClCa family
CNG: cyclic nucleotide-gated channel
Golf: GTP-binding protein of olfactory cilia
OSN: olfactory sensory neuron
